# Targeting the proteasome in epilepsy

**DOI:** 10.18632/oncotarget.18418

**Published:** 2017-06-08

**Authors:** Tobias Engel, Jose J. Lucas, David C. Henshall

**Affiliations:** Department of Physiology and Medical Physics, Royal College of Surgeons in Ireland, Dublin, Ireland

**Keywords:** status epilepticus, epilepsy, ubiquitin proteasome system, neuroprotection

Epilepsy is a group of neurological syndromes characterized by the occurrence of recurrent spontaneous seizures [1]. These arise due to transient imbalances between excitation and inhibition in the brain and may have a genetic origin or are acquired as a result of an insult to the brain. Epileptogenesis is the process whereby the brain undergoes pathological changes which eventually lead to the emergence of spontaneous seizures.

Epileptogenesis is associated with large-scale changes in protein expression which contribute to hyperexcitability-promoting alterations in neuronal networks and synaptic transmission [2]. While most focus has understandably been on transcriptional changes there is growing evidence that turnover of proteins within cells, particularly locally, at synapses, plays a critical role.

The ubiquitin proteasome system (UPS) is highly conserved among different species and is the principal intracellular pathway leading to the clearance of short-lived, aberrant and/or misfolded proteins. The UPS consists of two separate steps including protein labeling via polyubiquitination and the subsequent degradation of polyubiquitinated proteins by the proteasome [3]. An impairment of the UPS has been proposed as a common pathological characteristic among acute and chronic brain diseases including ischemia, traumatic brain injury, neurodegenerative diseases and epilepsy [3–5]. Consequently, modulators of proteasome function have been postulated as a potential new treatment strategy for these diseases [3].

Previous studies suggest UPS function is also altered in epilepsy and there is in vitro evidence that increased neuronal activity interferes with a correct functioning of the proteasome [6]. The immunoproteasome which plays an important role in the degradation of damaged proteins resulting from cytokine-stimulated protein synthesis during an inflammatory response or oxidative stress is also up-regulated in experimental epilepsy models and in the brain of epilepsy patients [5]. Whether UPS function is compromised during seizures in vivo, however, had not been explored. By using a UPS green-fluorescent protein (GFP)-reporter mouse we demonstrated that there is impairment of UPS function following prolonged seizures (status epilepticus) triggered by an injection of the excitotoxin kainic acid in mice. The UPS dysfunction persisted into the period when mice display recurrent spontaneous seizures [7]. However, the most striking finding was that UPS inhibition displayed sharp spatio-temporal changes as it evolved over time, being most evident in brain regions resistant to seizure-induced cell death. The same response was found when seizures were triggered by another drug, pilocarpine, ruling out this being an artifact of the specific model. This prompted us to ask whether proteasome inhibition might provide neuroprotective effects. To test this idea we treated mice with a potent and selective inhibitor just before status epilepticus and showed that damage to the hippocampus could be reduced [7]. Although surprising, others have reported similar benefits of this class of drug in other models, including stroke [4]. The mechanism of the protection is currently unknown but may result from accumulation of proteins that mediate synaptic reorganization or neuroinflammatory signaling [4]. Evidence for a synaptic link was given a boost by the recent demonstration by our group that loss of ubiquitin carboxyl-terminal hydrolase isozyme L1 (UCHL1), deubiquitinating enzyme, occurs during status epilepticus and pharmacologic inhibition of the enzyme reduced levels of a key post-synaptic protein and aggravated seizures [8].

There was another notable observation in our study. UPS impairment was first evident in the principal excitatory neurons of the hippocampus. However, UPS inhibition emerged in astrocytes at later stages [7]. We do not know why different cell populations undergo an impairment of the UPS at different time-points; however, it is tempting to speculate that this progression reflects specific metabolic or signaling changes that accompany responses to status epilepticus. Whereas neurons are recruited during acute seizures, astrocytes are activated during the inflammatory processes occurring at later disease stages.

Several conclusions can be drawn from our studies. (1) An impairment of the UPS possibly contributes to changes in the proteome following seizures and during epilepsy. (ii) UPS inhibition is dependent on cell type and brain area affected. (iii) UPS inhibition may be beneficial at a certain range, (iv) seizures are a frequent co-morbidity among other brain diseases, therefore the observed UPS impairment in these diseases might be in part due to increased hyperexcitability states, and (v), finally, the identification of proteins showing a reduction in their UPS-dependent degradation during seizures might lead to the discovery of new drug targets for epilepsy.

Our findings represent key steps in identifying the exact role the UPS carries out during seizures and epilepsy and helps point toward UPS-targeting drugs as potential novel treatments for epilepsy. Several unanswered questions remain, critical to further determine the therapeutic potential of UPS interference during epilepsy.

What are the molecules that accumulate during UPS inhibition after seizures and do these differ between acute and chronic stages? Second, proteasome inhibition was only effective at a specific dose. What is the exact dose window to protect the brain and when does it become neurotoxic? Last, does proteasome inhibition benefit or harm the epileptic brain?

In conclusion, inhibition of proteasome function may constitute an endogenous protective mechanism in the setting of prolonged seizures. While it is unlikely that UPS-interfering drugs will be used in the future to treat epilepsy, identifying proteins which are regulated by the proteasome during seizures may represent new drug targets to protect the brain from seizure-induced damage.
